# Loss, gain and choice difficulty in gambling patients: Neural and behavioural processes

**DOI:** 10.1111/adb.13396

**Published:** 2024-05-10

**Authors:** Daniel Freinhofer, Philipp Schwartenbeck, Natasha Thon, Wolfgang Aichhorn, Melanie Lenger, Friedrich M. Wurst, Martin Kronbichler

**Affiliations:** ^1^ Centre for Cognitive Neuroscience and Department of Psychology University of Salzburg Salzburg Austria; ^2^ Neuroscience Institute Christian‐Doppler Medical Centre Paracelsus Medical University Salzburg Salzburg Austria; ^3^ Wellcome Trust Centre for Human Neuroimaging University College London London UK; ^4^ Oxford Centre for Functional MRI of the Brain Nuffield Department of Clinical Neurosciences University of Oxford Oxford UK; ^5^ Department of Psychiatry, Psychotherapy and Psychosomatics Christian‐Doppler Medical Centre Paracelsus Medical University Salzburg Austria; ^6^ Department for Psychiatry and Psychotherapy Medical University of Graz Graz Austria; ^7^ Medical Faculty and Psychiatric University Hospital University Basel Basel Switzerland

**Keywords:** choice difficulty, decision‐making, fMRI, gambling, loss aversion

## Abstract

Impaired decision‐making is often displayed by individuals suffering from gambling disorder (GD). Since there are a variety of different phenomena influencing decision‐making, we focused in this study on the effects of GD on neural and behavioural processes related to loss aversion and choice difficulty. Behavioural responses as well as brain images of 23 patients with GD and 20 controls were recorded while they completed a mixed gambles task, where they had to decide to either accept or reject gambles with different amounts of potential gain and loss. We found no behavioural loss aversion in either group and no group differences regarding loss and gain‐related choice behaviour, but there was a weaker relation between choice difficulty and decision time in patients with GD. Similarly, we observed no group differences in processing of losses or gains, but choice difficulty was weaker associated with brain activity in the right anterior insula and anterior cingulate cortex in patients with GD. Our results showed for the first time the effects of GD on neural processes related to choice difficulty. In addition, our findings on choice difficulty give new insights on the psychopathology of GD and on neural processes related to impaired decision‐making in GD.

## Graphical abstract

We recorded behavioural responses and brain images of patients with gambling disorder (GD) and controls while they completed a mixed gambles task. We found no group differences in loss‐ and gain‐related choice behaviour, but choice difficulty was stronger associated with decision time in controls than in GD patients. Similarly, we observed no group differences in processing of losses or gains, but choice difficulty and brain activity in the right anterior insula were stronger associated in controls than in GD patients.

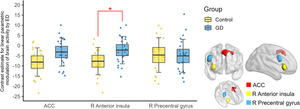

## 1 INTRODUCTION

Individuals suffering from pathological gambling or gambling disorder (GD) often make bad decisions — not only in gambling situations but also in everyday life situations and lifestyle choices.^1^ This impairment of decision‐making is reflected in the characterizations of GD in the ICD‐11^2^ as well as in the DSM‐5.^3^ Decision‐making is a complex cognitive process with a variety of different phenomena influencing how individuals make choices. In this study, we focused on the influence of GD on neural and behavioural processes related to loss aversion and choice difficulty.

### 1.1 Loss aversion

Loss aversion describes the tendency that people experience more satisfaction from avoiding losses than they experience satisfaction from acquiring gains of an equivalent value.^4^ Both particularly high and low levels of loss aversion are related to poor decision‐making and are associated with mental disorders. Increased loss aversion is associated with obsessive compulsive disorder,^5^ whereas reduced loss aversion is associated with substance‐use disorders.^6^ Since GD is closely related to substance‐use and other addiction disorders,^3^ one would assume that patients with GD also exhibit reduced loss aversion, but the literature shows diverging results. On the one hand, multiple studies reported reduced loss aversion in GD,^7^, ^8^, ^9^ which would be in line with a typical symptom of GD, namely the tendency to continue a certain behaviour despite negative consequences. On the other hand, some studies reported similar loss aversion in GD and control groups^10^ or even increased loss aversion for gambling patients who were in clinical treatment.^11^ As our first research question, we examined how GD influences loss aversion and hypothesized that gambling patients show reduced loss aversion since the majority of studies showed this effect.

Functional magnetic resonance imaging (fMRI) studies showed neural correlates of loss aversion in the striatum and ventromedial prefrontal cortex^12^ as well as in the amygdala, thalamus, and insula.^13^ Investigating loss aversion in subjects suffering from GD and alcohol use disorder, Genauck et al.^9^ revealed findings on altered functional connectivity between amygdala and prefrontal areas in subjects with GD, but no other differences between controls and subjects with GD in brain activity associated with losses or gains. Our second research question is concerned with the effect of GD on neural processes of losses and gains. Focusing on the striatum, which is related with loss aversion^9^, ^12^, ^13^, ^14^ and GD,^15^, ^16^ we hypothesized a weaker modulation of striatum activity by losses in gambling patients.

### 1.2 Choice difficulty

A choice between two possibilities can be easy, when one of those possibilities is appealing and the other one is unappealing. When both possibilities are comparatively appealing or comparatively unappealing though, it would be considered a harder choice.^17^ In other words, the more similar two options of a choice, the higher the choice difficulty. Time needed to make a decision is related to choice difficulty, with easier choices associated with a shorter response time (RT). This association was reported for various decision‐making tasks, where choice difficulty varied trial‐by‐trial depending on different outcomes and payout delays,^18^ depending on different outcomes and winning probabilities^19^ or depending on different values for potential gains and losses.^20^ Regarding GD or GD‐related disorders, we found only one study, which reported no effect of alcohol use disorder on the relation between choice difficulty and RT.^18^ However, Heeren et al.^20^ revealed a smaller RT difference between easy and hard choices in participants, who exhibit lower loss aversion. Considering that reduced loss aversion could be associated with GD, this would indicate a weaker influence of choice difficulty on RT in gambling patients. Thus, for our third research question, we tested if patients with GD exhibit a reduced effect of choice difficulty on RTs.

Regarding the neural processing of choice difficulty, fMRI studies reported stronger activation in the anterior cingulate cortex (ACC) for harder choices compared with easier choices, both for low level perceptual decision‐making^21^ as well as for higher level cognitive decision‐making.^22^, ^23^ This is in line with the proposed function of the ACC as a monitor that detects conflict between mutually exclusive alternatives.^24^ Other brain regions related to choice difficulty include the orbitofrontal cortex, the right inferior frontal gyrus and the anterior insula.^22^ Concerning the effects of GD on neural responses to choice difficulty, one study used choice difficulty as a covariate for analysing loss aversion in a GD sample, but they did not investigate neural processing of choice difficulty.^9^ Following our assumption, that GD reduces the influence of choice difficulty on RT, and in line with GD showing decreased brain activity in the ACC during choice‐related processes,^25^ we tested as our final research question, if choice difficulty is weaker associated with brain activity in the ACC in gambling patients.

### 1.3 Summary of aims & hypotheses

In the current study, we used a mixed gamble task during fMRI recording, to investigate neural and behavioural processes related to loss aversion and choice difficulty in gambling patients. We hypothesized that GD would be accompanied by reduced loss aversion as well as a weaker influence of choice difficulty on RTs. With regard to neural processes, we predicted that gambling patients would exhibit a weaker modulation of neural activity in the striatum by losses, and a weaker modulation of neural activity in the ACC by choice difficulty.

## 2 METHODS

### 2.1 Participants and assessment

Twenty‐six participants diagnosed with pathological gambling according to the DSM‐IV^26^ (which corresponds with GD in the newer DSM‐5) and 23 controls without history of neurological or mental disorders, or history of gambling behaviour initially took part in this study. After excluding six participants (see Results section), the final sample included 23 patients with GD and 20 controls. Sample size was chosen based on sample sizes of previous studies. Participants of the GD group were recruited from the Christian–Doppler Medical Centre in Salzburg, where they were treated for GD. Control participants were matched for sex and age, and were recruited by word‐of‐mouth advertising and through mailing lists. All participants had normal or corrected‐to‐normal visual acuity.

Questionnaires used in this study included the Alcohol Use Disorders Identification Test,^27^ the Fagerstrom Test for Nicotine Dependence,^28^ the State Anxiety Inventory,^29^ the Becks Depression Inventory (BDI,^30^) and the South Oaks Gambling Screen (SOGS,^31^).

This study has been approved by the local ethics committee (ethics committee of the Federal State Salzburg, Number 415‐E/1632/9‐2013). All participants provided informed written consent to take part in the study after being informed about potential risks of MRI recordings.

### 2.2 Experimental design

**FIGURE 1:**
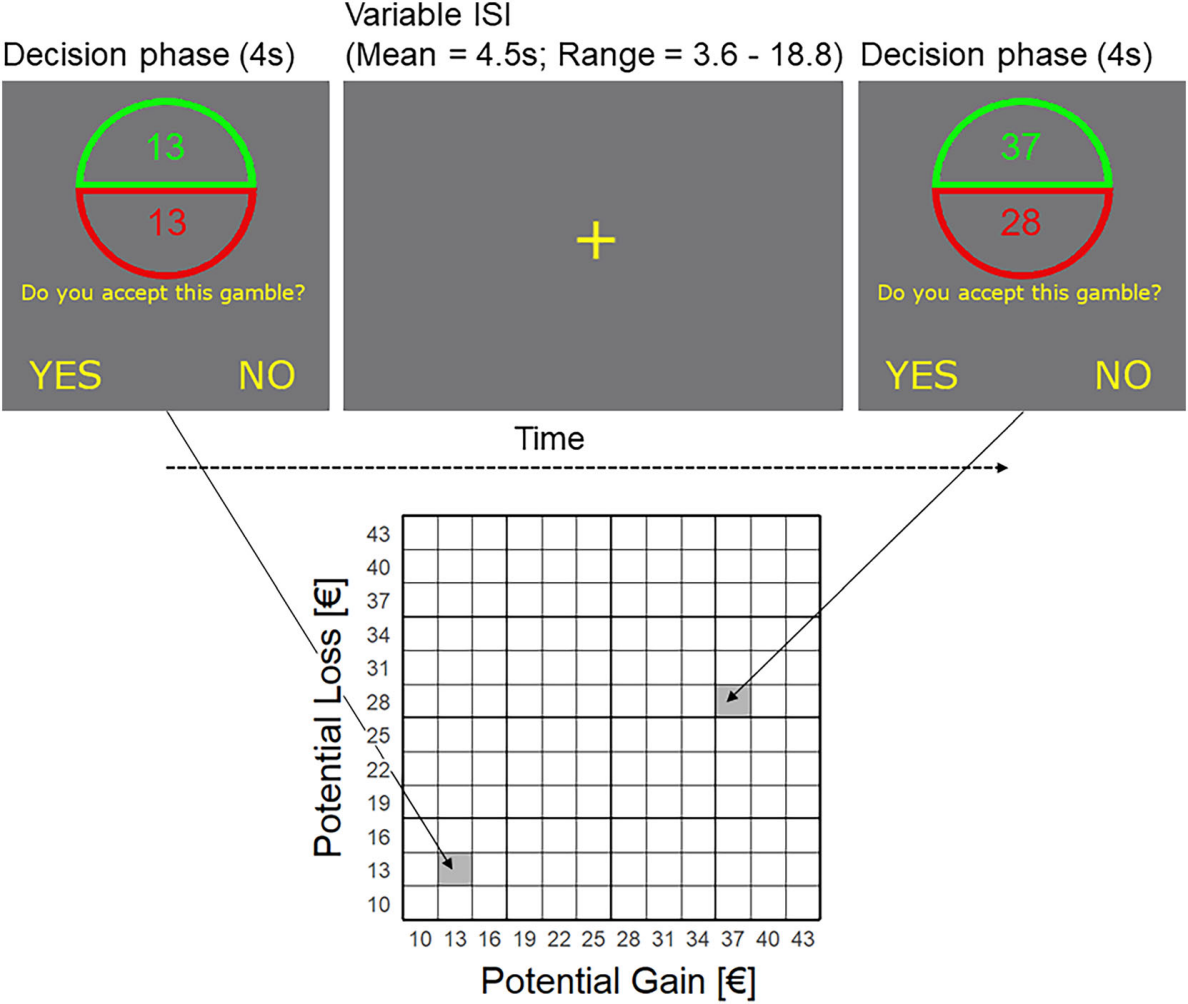
Mixed gamble task. Participants were asked to accept or reject gambles with equal probability to gain or lose different amounts of money. Gains (upper green semicircle) and losses (lower red semicircle) were sampled from an equal range of values ranging from 10€ to 43€.

During the mixed gambles task (adapted from^12^), gambles consisting of potential gains and losses were presented (see Figure 1). Participants were informed, that every gamble would have an equal probability for winning or losing, and they had to indicate if they would accept or reject to play the displayed gamble by pressing a left or right button during a 4‐second long decision phase. The next gamble was presented after a variable interstimulus interval (mean = 4.5 s). Because of ethical concerns with exposing patients with GD to real gambling situations, we could only use hypothetical gambles and participants could not win or lose real money. The task consisted of 144 gambles using every possible combination of gains and losses by sampling from 12 equidistant levels of an equal range of values ranging from 10€ to 43€.

MRI recording lasted approximately 1 h and included two 11‐minute fMRI sessions of 72 gambles each with a self‐paced break in between for the mixed gambles task as well as a structural scan and several other fMRI sessions, which are not reported here. Additionally, participants completed decision‐making tasks outside the MRI, which were reported in a previous publication.^32^

### 2.3 MRI acquisition

Brain images were recorded with a 3 Tesla Siemens Tim Trio MRI scanner using a 32‐channel head coil. Functional data was acquired with T2*‐weighted echo planar imaging sequences, TR = 2,520 ms, TE = 35 ms, flip angle = 77°, field of view = 192 × 192 mm, plane resolution = 2 × 2 mm, slice thickness = 3 mm, 36 slices and a slice gap of 0.3 mm. We acquired 252 volumes per session of the mixed gambles task including six initial dummy scans before stimulus presentation. Structural scans were acquired using a sagittal T1‐weighted MPRAGE sequence with following parameters: TR = 2,300 ms; TE = 2.91 ms; voxel size = 1.2 × 1 × 1 mm; slice thickness = 1.2 mm, field of view = 240 × 256 mm; 160 slices, flip angle = 9°.

### 2.4 Data analysis

#### 2.4.1 Behavioural data

R* was used for statistical analysis of behavioural data. After discarding responses with RT smaller than 100 ms or bigger than 4000 ms, a logistic regression was performed separately for every participant with potential loss and gain as independent variables and choice (acceptance or rejection) as dependent variable. We calculated the loss aversion parameter λ as:

$$\lambda =\frac{-\beta_{\text{Loss}}}{\beta_{\text{Gain}}}$$

where *β*_*Loss*_ and *β*_*Gain*_ are the unstandardized regression coefficients for potential loss and gain, respectively. Following Tom et al.,^12^ we simplified the calculation of λ with assumptions of a linear rather than a curvilinear value function and did not include a probability weighting function. A value of λ > 1 indicates loss averse behaviour, whereas λ < 1 indicates loss prone behaviour. *t*‐Tests were used to investigate group difference in λ and overall RTs. As a measure of choice difficulty, we calculated the Euclidean distance (ED) of each gamble's gain and loss value from the diagonal of gambles with equal gains and losses. ED ranged from 0 to 23.33, with 0 indicating hardest choices (equal loss and gain) and the maximum of 23.33 indicating easiest choices (largest difference between loss and gain). To compute the main effect for ED and the interaction of group and ED we used a linear mixed‐effects model of the *nlme* package^33^ with RT as dependent variable, group and ED as fixed effects, and subjects including random slopes for ED as random effects.

#### 2.4.2 Neuroimaging data

Preprocessing and statistical analysing of neuroimaging data was conducted with SPM12† running in a Matlab R2013a environment (Mathworks Inc.), with addition of functions from AFNI.‡ After discarding the first six dummy scans, preprocessing of functional images included realignment, de‐spiking, unwarping, correction for geometric distortions using individual fieldmaps, and slice‐time correction. High resolution T1‐weighted images were processed via the CAT12 toolbox§ by segmenting structural images of each participant into grey matter, white matter, and CSF. After denoising the segmented images, we registered them to a DARTEL template provided by the CAT12 toolbox via the high dimensional DARTEL registration algorithm,^34^ to warp each image into MNI space and obtain a skull stripped version of each image in native space. Functional images were normalized to MNI space by coregistration to the skull striped structural image and warping the functional images with the parameters from the DARTEL registration. Finally, functional images were resampled to 3 × 3 × 3 mm voxels and smoothed with a 6 mm FWHM Gaussian kernel.

Statistical analysis was performed with a general linear model (GLM) two‐staged mixed effects model. For the subject‐specific first level model, we combined both sessions of the task and modelled each gamble by convolving a stick function at its onset with a canonical hemodynamic response function of SPM12. Gain, loss and ED were z‐transformed and modelled by convolving a proportionally sized stick function at gamble onset with the same canonical hemodynamic response function. Gain and loss were combined in one first level model, whereas ED was analysed in a separate first level model. The default orthogonalization of SPM was deactivated for this analysis. Six motion regressors obtained from the realignment process, a regressor representing gambles with missing responses and a regressor indicating the respective session were modelled as regressors of no interest. Contrast estimates of the linear parametric modulation of brain activity by gain, loss, the difference between gain and loss (gain > loss), and ED were computed for every subject with the first‐level GLMs using temporal high pass filtering (cut off frequency = 1/128 Hz) to remove low‐frequency drifts and modelling temporal autocorrelation with an AR(1) process.^35^

For whole‐brain group‐level analyses, we rescaled the first‐level contrast images by vascular auto‐rescaling of fMRI technique^36^ to decrease interindividual variability and increase statistical sensitivity. We performed second‐level analyses for each of the rescaled contrasts using ANOVAs with group (GD vs. control) as between‐group factor and separate one‐sample *t*‐tests for controls and GD group. Additionally, we used the gain > loss contrast and the loss aversion parameter λ to analyse the correlation between behavioural loss aversion and neural loss aversion.^12^ Since patients with GD had significant higher depression scores than controls, we included BDI scores as a covariate of no interest in all second‐level analyses. All whole‐brain results are reported with an uncorrected voxel‐level threshold of *p* < 0.001 and a family‐wise error (FWE) corrected cluster‐level threshold of *p* < 0.05.

For region of interest (ROI) analyses, we extracted contrast estimates of the three effects of interest (gain, loss and ED) for each participant across all voxels within three different sets of ROIs. *t*‐Tests were computed with these ROI estimates to investigate group differences. To define ROIs we exported false discovery rate corrected (*p* < 0.01) contrast images of automated meta‐analyses created by *neurosynth.org* ^37^ using the search terms “Losses”, “Gains”, and “Difficulty” and excluded all above‐threshold clusters with voxel extent *k* < 50 (see Figure 4 for a depiction of the resulting ROIs). For “Losses”, 95 studies were aggregated and resulting ROIs included right striatum (*k* = 172, centre = [12, 8, 1]), left striatum (*k* = 86, centre = [−9, 2, 4]) and medial superior frontal gyrus (*k* = 56, centre = [−3, 35, 31]). For “Gains”, 137 studies were aggregated and resulting ROIs included right striatum (*k* = 140, centre = [18, 12, 2]) and left striatum (*k* = 103, centre = [−14, 11, 1]). For “Difficulty”, 410 studies were aggregated and resulting ROIs included ACC (*k* = 108, centre = [−8, 12, 46]), right anterior insula (*k* = 69, centre = [34, 24, 0]) and right precentral gyrus (*k* = 67, centre = [45, 8, 28]).

## 3 RESULTS

Six of initial 49 participants had to be excluded from the study either because of high SOGS score in control participants or because of inadequate response patterns. One control participant did not fulfil the requirements for the control group because they had a SOGS score of 5. Two participants rejected every gamble, which makes it unfeasible to calculate a loss aversion parameter. Three participants displayed inexplicable response patterns, indicating that either lower gains or higher losses lead to higher probability of accepting gambles, which suggests that these participants either did not understand the task or responded unreasonably on purpose (see Supplementary Figure S1). Sample characteristics of the remaining 43 participants are shown in Table 1. There was no significant group difference in age, tobacco consumption and alcohol consumption. The GD group had fewer average years of education, exhibited increased depression and state anxiety scores, and showed higher gambling scores. All GD participants were in clinical treatment for GD and duration since their last gambling behaviour ranged from 2 to 32 months, with a median of 8 months.

**TABLE 1:** Sample characteristics for control and GD group.

	Control group	GD group	Test statistics/group differences
N/female	20/2	23/3	*OR* = 0.75, *p* = 1.000
Age	38.55 (12.88)	43.04 (12.02)	*t*(41) = −1.18, *p* = 0.24, *d* = 0.36
Age range	21–60	22–63	
Years of Education	11.32 (1.42)	9.68 (1.46)	*t*(39) = 3.62, *p* = 0.00, *d* = 1.13*
FTND	0.90 (1.68)	1.86 (2.14)	*t*(40) = −1.61, *p* = 0.12, *d* = 0.50
AUDIT	3.70 (2.54)	4.56 (5.64)	*t*(36) = −0.61, *p* = 0.54, *d* = 0.20
BDI	5.10 (5.54)	13.95 (12.90)	*t*(29.07) = −2.94, *p* = 0.01, *d* = 0.89*
STAI	35.75 (8.90)	44.90 (13.63)	*t*(35) = −2.33, *p* = 0.03, *d* = 0.77*
SOGS	0.16 (0.50)	9.86 (3.24)	*t*(22.16) = −13.86, *p* < 0.001, *d* = 4.19*

*Note*: Values in parentheses represent standard deviation.

Abbreviations: FTND, Fagerström Test for nicotine dependence; AUDIT, Alcohol Use Disorders Identification test; BDI, Beck Depression Inventory; STAI, State version of the State Rate Anxiety Inventory; SOGS, South Oaks Gambling Scale.

* *p* < 0.05.

### 3.1 Behavioural results

**FIGURE 2:**
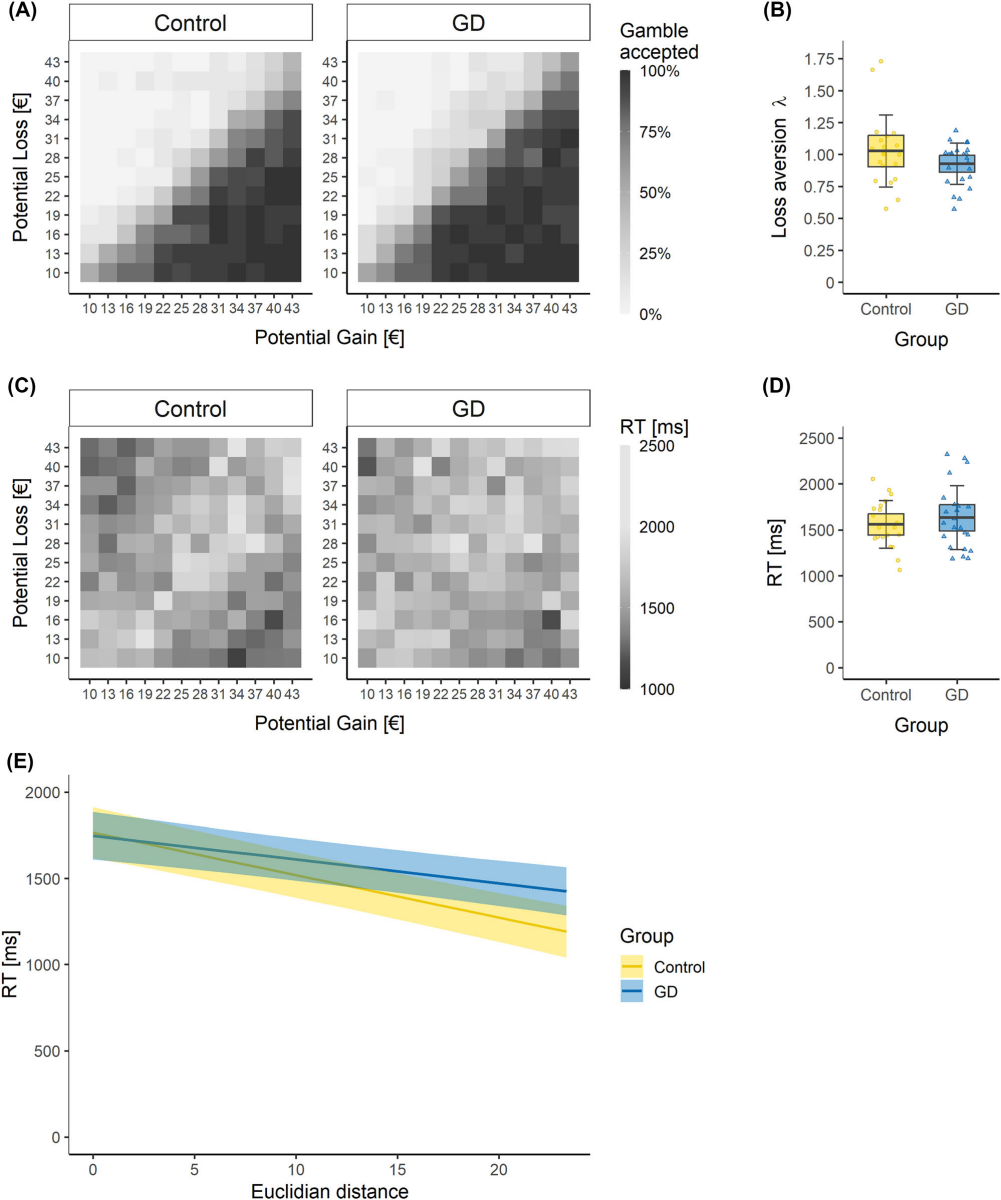
(A) Heatmaps showing average acceptance rate of every gamble. (B) Average loss aversion. (C) Heatmaps showing average RT of every gamble. (D) Average RT. (E) Relation between RT and ED. Data points indicate individual participants. Thick red horizontal line = mean; shaded regions *±* error bars = 95 confidence intervals *±* 1 s.d. of the mean.

Both groups had similar response patterns with regard to gamble acceptance (see Figure 2A) and percentage of missing response (ranging from 0% to 17%, with no group difference, χ2 = 0.95, *p* = 0.33). We found no significant difference between controls (λ = 1.03) and GD group (λ = 0.93) in loss aversion (t[29.40] = 1.40, *p* = 0.170, d = 0.43, see Figure 2B). One sample *t*‐tests revealed no significant loss aversion (λ > 1) neither in controls (t[19] = 0.42, *p* = 0.340) nor in the GD group (t[22] = −2.18, *p* = 0.980).

Controls (mean = 1,558 ms) and patients with GD (mean = 1,631 ms) did not significantly differ in RT (*t*(41) = −0.77, *p* = 0.450, *d* = 0.23, see Figure 2D). Also, RTs did not differ between accepted and rejected gambles neither in controls (*p* = 0.947) nor in the GD group (*p* = 0.903). The linear mixed‐effects model with RT as dependent variable and group and ED as fixed factors showed a significant main effect for ED (*t* = −8.17, *p* < 0.001) and a significant interaction between group and ED (*t* = 2.62, *p* = 0.009), indicating that responses were faster for gambles with higher ED, but this relation between ED and RT was weaker in gambling patients (see Figure 2C & E). Since BDI scores were included as a covariate in neuroimaging analyses, we also checked the behavioural results with BDI as a covariate and found no significant changes in the results.

### 3.2 Neuroimaging results

**FIGURE 3:**
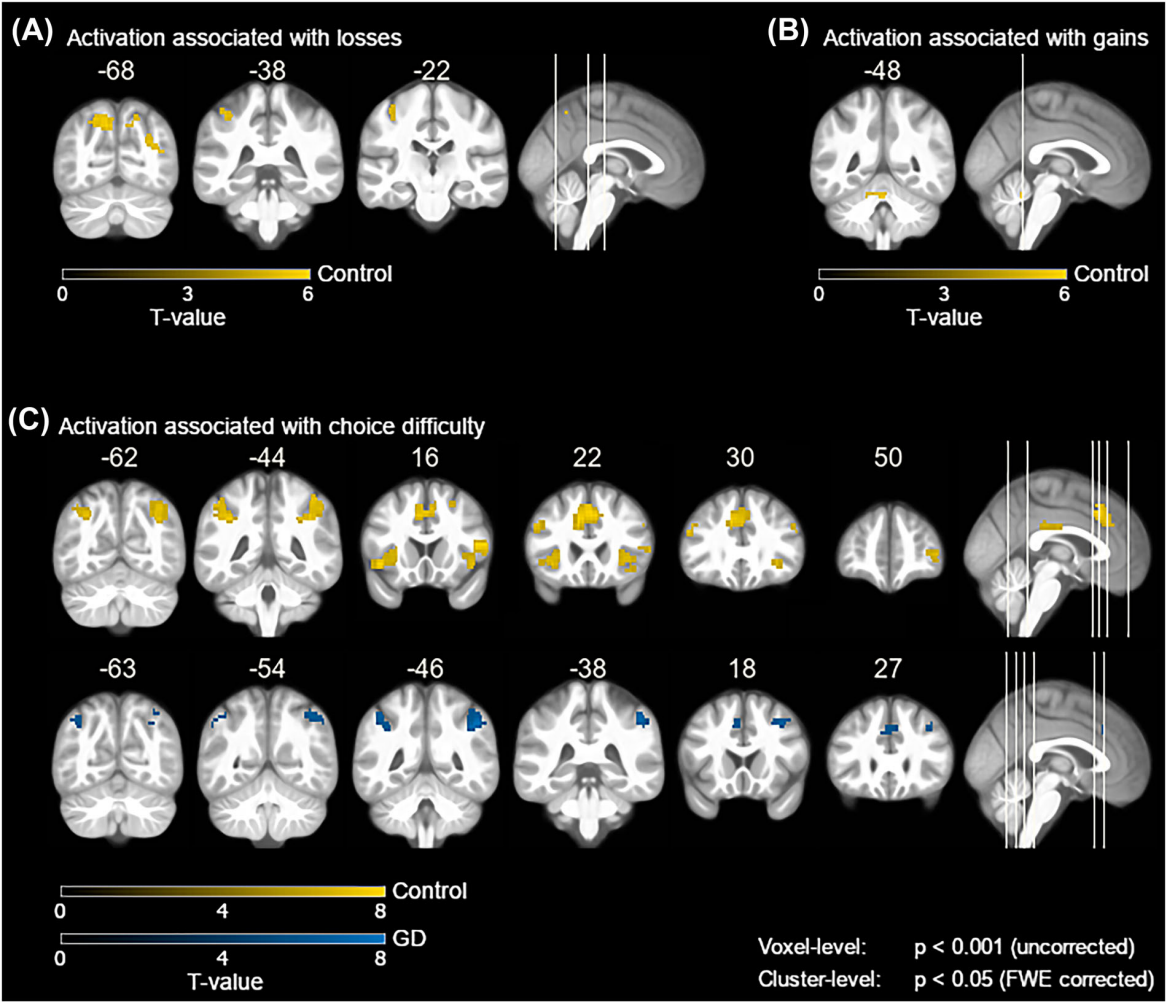
(A) Brain regions showing neural activity modulated by magnitude of losses. (B) Brain regions showing neural activity modulated by magnitude of gains. (C) Brain regions showing neural activity modulated by magnitude of choice difficulty.

**FIGURE 4:**
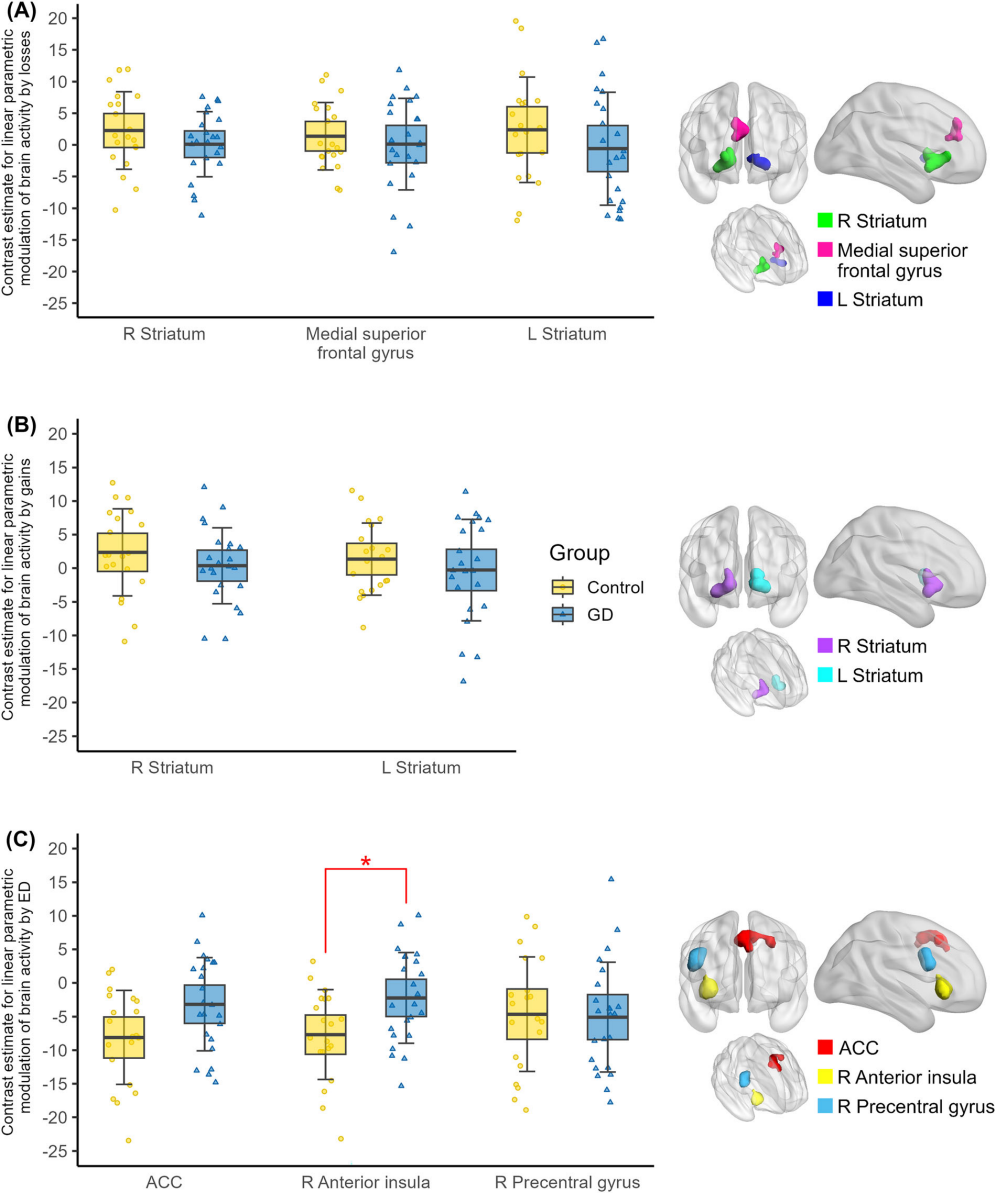
ROI analysis for (A) losses, (B) gains, and (C) ED (inverse measure for choice difficulty). Data points indicate individual participants. Thick red horizontal line = mean; shaded regions *±* error bars = 95 confidence intervals *±* 1 s.d. of the mean for each group. * *p* < 0.05 for two‐sample *t*‐test (two‐tailed).

Whole‐brain analyses showed no significant group difference in brain activity modulated by gains or losses (see T maps for all whole‐brain contrasts at https://neurovault.org/collections/IVFFLGPF/), and there were also no group differences in the ROI analyses (*ps ≥* 0.46, see Figure 4A and Figure 4B). Separately for controls, we found larger losses associated with higher activity in occipital and parietal regions (see Table 2 and Figure 3A), and larger gains associated with higher activity in the cerebellum (see Table 2 and Figure 3B). For the GD group, we found no significant modulation of neural activity by gains or losses. There was no group difference in brain activity modulated by the gain > loss contrast, and there was no correlation between the behavioural loss aversion parameter (λ) and neural loss aversion (gain > loss contrast).

**TABLE 2:** Neural activity modulated by magnitude of gains or losses.

		MNI			Peak	Cluster
Cluster size (voxels)	Anatomical region	*x*	*y*	*z*	*T* score	*p* value (FWE‐corr)
Losses ‐ Control group						
139	L Precuneus	−9	−70	43	5.01	<0.001
	L Precuneus	−3	−58	55	4.35	
	L Superior parietal lobule	−18	−67	49	4.31	
51	R Middle occipital gyrus	36	−70	25	4.72	0.004
	R Superior occipital gyrus	27	−70	28	4.52	
	R Superior parietal lobule	30	−61	31	3.69	
56	L Postcentral gyrus	−36	−25	49	4.45	0.003
	L Postcentral gyrus	−39	−37	55	4.06	
	L Postcentral gyrus	−39	−34	46	3.76	
56	R Precuneus	15	−58	37	4.08	0.003
	R Superior parietal lobule	21	−61	52	3.83	
	R Precuneus	21	−52	34	3.63	
Gains ‐ Control group						
39	L Cerebellum	−6	−55	−20	4.95	0.019
	L Cerebellum	0	−49	−23	4.42	
	L Cerebellum	−9	−64	−20	4.39	

*Note*: L = Let; R = Right.

For modulation by ED, we found no group difference on the whole‐brain level, but ROI analyses revealed a stronger negative modulation of brain activity by ED in controls compared with gambling patients. Thus, larger choice difficulty was stronger associated with higher neural activity in controls compared with patients with GD in the right anterior insula (*t*(41) = −2.77, *p* = 0.008, *η* ^*2*^_*p*_ = 0.15, see Figure 4C), and there was a smaller, almost significant effect in the ACC (*t*(41) = −1.88, *p* = 0.068, *η* ^*2*^_*p*_ = 0.12). Separate whole‐brain analyses for ED revealed associations between larger choice difficulty and higher brain activity in the ACC, the superior frontal gyrus, the supplementary motor area, the left and right anterior insula, the right middle frontal gyrus, and several other frontal and parietal areas for controls; And in the left and right supramarginal gyrus, right middle frontal gyrus and other frontal areas for the GD group (see Table 3 and Figure 3C).

**TABLE 3:** Neural activity modulated by magnitude of choice difficulty.

		MNI			Peak	Cluster
Cluster size (voxels)	Anatomical region	*x*	*y*	*z*	*T* score	*p* value (FWE‐corr)
Control group						
233	L Anterior cingulate gyrus	−9	26	34	7.20	<0.001
	R Supplementary motor area	3	23	40	6.38	
	L Supplementary motor area	−6	20	40	5.84	
176	R Inferior frontal gyrus	51	17	13	6.65	<0.001
	R Anterior insula	33	20	1	5.27	
	R Anterior insula	33	29	−5	4.89	
240	L Supramarginal gyrus	−45	−46	52	6.37	<0.001
	L Superior parietal lobule	−33	−49	40	6.14	
	L Angular gyrus	−39	−55	49	5.45	
113	L Anterior insula	−42	17	−5	6.09	<0.001
	L Anterior insula	−27	20	4	5.55	
	L anterior insula	−33	23	−5	5.06	
343	R Angular gyrus	33	−58	40	5.91	<0.001
	R Angular gyrus	33	−67	49	5.80	
	R Superior parietal lobule	39	−43	37	5.50	
46	L Middle frontal gyrus	−45	26	31	5.25	0.005
	L Middle frontal gyrus	−48	35	19	4.66	
80	R Middle frontal gyrus	45	35	19	5.08	<0.001
	R Middle frontal gyrus	45	41	13	5.00	
	R Middle frontal gyrus	42	50	4	4.49	
33	R Superior frontal gyrus	24	11	49	5.00	0.024
34	L Middle cingulate gyrus	−3	−16	28	4.24	0.021
	L Posterior cingulate gyrus	−3	−28	31	4.23	
	L Posterior cingulate gyrus	−3	−37	28	3.64	
GD group						
189	R Supramarginal gyrus	48	−43	43	7.55	<0.001
	R Supramarginal gyrus	48	−37	49	5.95	
	R Angular gyrus	39	−55	52	4.71	
84	L Supramarginal gyrus	−42	−49	49	5.47	<0.001
	L Angular gyrus	−39	−64	49	4.76	
	L Angular gyrus	−36	−49	40	4.46	
60	R Middle frontal gyrus	36	17	43	5.00	0.001
	R Middle frontal gyrus	39	26	40	4.42	
	R Precentral gyrus	42	−1	31	4.16	
29	L Supplementary motor area	−6	17	40	3.93	0.040
	L Superior frontal gyrus	−3	29	40	3.90	

*Note*: L = Left; R = Right.

## 4 DISCUSSION

We assumed that gambling patients exhibit reduced loss aversion and that their decision‐making is less affected by choice difficulty. Results showed no loss aversion in controls or patients with GD and no differences in the overall choices, but choice difficulty did affect RTs less in patients with GD. Additionally, we examined how GD affects neural responses to losses, gains and choice difficulty. We observed no group differences in processing of losses or gains, but choice difficulty was weaker associated with brain activity in the right anterior insula and the ACC in patients with GD.

### 4.1 (No) loss aversion

Before we discuss the influence of GD on decision‐making, we must first address the elephant in the room: In this study, we wanted to explore differences in loss aversion between gambling patients and controls, but our results showed no significant loss aversion (λ > 1) emerging in either group.

One explanation for the absence of loss aversion could be, that modifications of the experiment in our study changed participants' perception of the potential losses and gains. In contrast to most studies with similar mixed gambles tasks, we did not use real money as incentives. Consequently, participants did not receive an initial payment nor did they receive money depending on their choices.^7^, ^9^, ^11^, ^12^ Tasks with hypothetical incentives usually show outcomes comparable with tasks with monetary incentives^38^ and our participants exhibited overall reasonable choice behaviour (they preferred gambles with higher gains and lower losses), but we cannot rule out, that the changes in the experiment could have affected the emergence of loss aversion.

Recent reviews may provide additional insights on this “lack” of loss aversion. One review characterized loss aversion as dependent on context factors rather than a fundamental psychological bias.^39^ For example, if the absolute amount of losses is not large enough or if avoiding losses is presented as an active option that disturbs the status quo, no loss aversion occurs. In line with a meta‐analysis which reported a comparatively small average magnitude of loss aversion,^40^ these findings indicate that we maybe should challenge our conceptualization of loss aversion.^41^

However, even though both groups showed no loss aversion, it is still notable, that choice behaviour dependent on potential gains and losses is not altered in patients with GD. It would have been possible, that gambling patients not only show no loss aversion but in fact exhibit loss prone behaviour, where individuals are more sensitive to gains than to losses. But in line with Takeuchi et al^10^ and Giorgetta et al.,^11^ and in contrast with Brevers et al.,^7^ Gelskov et al.^8^ and Genauck et al.,^9^ patients with GD made choices similar to controls. An explanation could be that participants in our gambling group were treated for GD, and individuals with GD might behave differently in gambling situations after starting clinical treatment^11^, ^42^ compared with individuals with untreated GD.^7^

Additionally, we investigated if GD affects neural processing of gains and losses. In line with our behavioural findings, we found no alterations in neural responses to gains or losses in patients with GD. These results conform with findings by Genauck et al.,^9^ who reported loss‐related alterations of brain activity in alcohol dependent subjects but none in subjects with GD. In contrast to Tom et al.,^12^ we found no modulation of brain activity by gains in reward‐related areas like the striatum neither in the control nor in patients with GD. Since the amount of potential gains was not related to activity in reward‐related regions, participants apparently perceived the gains as less rewarding or desirable as participants in other studies. This would support the assumption, that the absence of loss aversion was connected to the changes in our experiment, which could have caused participant's to perceive gains and losses differently.

### 4.2 Choice difficulty

Choice difficulty had a weaker influence on decision‐making time in patients with GD, and we have several explanations, how GD could affect the role of choice difficulty in decision‐making: First, increased impulsivity in gambling patients, especially non‐planning impulsivity,^32^ could lead to decision‐making without considering all useful information including choice difficulty. Secondly, patients with GD could have different decision‐making strategies.^43^ They could, for example, only consider if the potential gain is bigger than the potential loss, but ignore the difference between gain and loss, which would reduce the influence of choice difficulty on decision‐making time. A third explanation could be, that GD biases the perception of choice difficulty.^44^ If patients with GD would perceive less difference between easy and hard decisions, this distinction should also have less influence on their RTs.

Future studies could test which of these explanations might best describe the effect of GD on processing of choice difficulty by asking participants after every gamble, how difficult this decision was. This additional measure could show, if patients with GD have different subjective estimations of choice difficulty or if they have similar estimations but do not consider choice difficulty in their decision process. Adding this question could also indicate, if RTs of gambling patients would be influenced more by choice difficulty, when choice difficulty is made explicit.

One would assume, that having choice difficulty less involved in the decision‐making process would lead to impaired decision‐making by not taking enough time for difficult decisions. On the contrary, GD only affected the influence of choice difficulty on RTs, but not the overall choices. This matches the results of our other study on GD, which reported no differences in overall choices, but a different evaluation of gambles in patients with GD.^32^ Together, these findings indicate, that even when choices appear unaffected, underlying decision‐making processes are altered in patients with GD.

Regarding neural processes, we found a weaker relation between choice difficulty and activity in the right anterior insula and the ACC in patients with GD. The right anterior insula is involved in decision‐making as a monitor of interoceptive information of body states and emotions,^45^ which aids evaluation of different options.^46^ Impaired functioning of the anterior insula is connected to decision‐making deficits like an insensitivity to value differences.^47^ Moreover, gambling severity is correlated with activity in the anterior insula.^48^ In line with these findings, our results suggest, that the anterior insula is important for processing of choice difficulty, and that this function of the anterior insula is impaired in patients with GD.

The ACC, which showed a smaller effect regarding choice difficulty, plays a central role in decision‐making and several functions have been attributed to it: generating voluntary choices, keeping track of outcomes and rewards, updating internal models of changing environments, and monitoring of choice difficulty.^23^, ^49^ In GD subjects, the ACC exhibits diminished activity during decision‐making,^25^ and gambling severity is related to higher serotonin levels in the ACC.^50^ In line with these findings, our results could indicate that the ACC's ability to monitor choice difficulty is impaired in gambling patients.

## 5 CONCLUSION

GD affected the influence of choice difficulty on decision time but had no effect on loss‐ and gain‐related choice behaviour. Furthermore, neural response to choice difficulty in gambling patients was reduced in the right anterior insula and in the ACC. To our knowledge, this is the first study reporting effects of GD on neural responses to choice difficulty. Thus, our findings give new insights on the psychopathology of GD and on neural processes related to impaired decision‐making in GD.

## AUTHOR CONTRIBUTIONS

Daniel Freinhofer drafted the original manuscript and carried out data analyses. Friedrich M. Wurst, Natasha Thon, and Wolfgang Aichhorn were responsible for diagnosing and recruiting participants. Melanie Lenger, Philipp Schwartenbeck, and Martin Kronbichler were responsible for data collection. Daniel Freinhofer and Martin Kronbichler participated in result interpretation and discussion, and in successive revisions of the original manuscript. Martin Kronbichler and Friedrich M. Wurst are the principal investigators of the research project.

## CONFLICT OF INTEREST STATEMENT

The authors declare no conflict of interest.

## ETHICS APPROVAL STATEMENT

This study has been approved by the local ethics committee (ethics committee of the Federal State Salzburg, Number 415‐E/1632/9‐2013).

## PATIENT CONSENT STATEMENT

All participants provided informed written consent to take part in the study after being informed about potential risks of MRI recordings.

## Supporting information


**Figure S1.** Individual response patterns for all 49 participants, which initially took part in the study. Because of our exclusion criteria, following participants were excluded from further data analysis: c08 (control with SOGS greater than 4), c15 and gd27 (rejected every gamble ‐unfeasible to calculate a loss aversion parameter), c10, gd09 and gd14 (inexplicable response patterns suggest these participants either did not understand the task or did response unreasonably on purpose)

**Data S2** Supporting information

**Figure S1.** Individual response patterns for all 49 participants, which initially took part in the study. Because of our exclusion criteria, following participants were excluded from further data analysis: c08 (control with SOGS greater than 4), c15 and gd27 (rejected every gamble ‐unfeasible to calculate a loss aversion parameter), c10, gd09 and gd14 (inexplicable response patterns suggest these participants either did not understand the task or did response unreasonably on purpose)

## Data Availability

The group data that support the findings of this study are openly available in NeuroVault at https://neurovault.org/collections/IVFFLGPF/. Data of individuals are not available because of privacy/ethical restrictions.

## Footnotes

*
https://www.r-project.org/.

†
http://www.fil.ion.ucl.ac.uk/spm/software/spm12/.

‡
https://afni.nimh.nih.gov/.

§
http://dbm.neuro.uni-jena.de/cat.
