# Supplementary figures and images for: Loss, gain and choice difficulty in gambling patients: Neural and behavioural processes
Source: Addict Biol. 2024 May 10;29(5):e13396. doi: 10.1111/adb.13396 (PMC11087675; doi:10.1111/adb.13396)

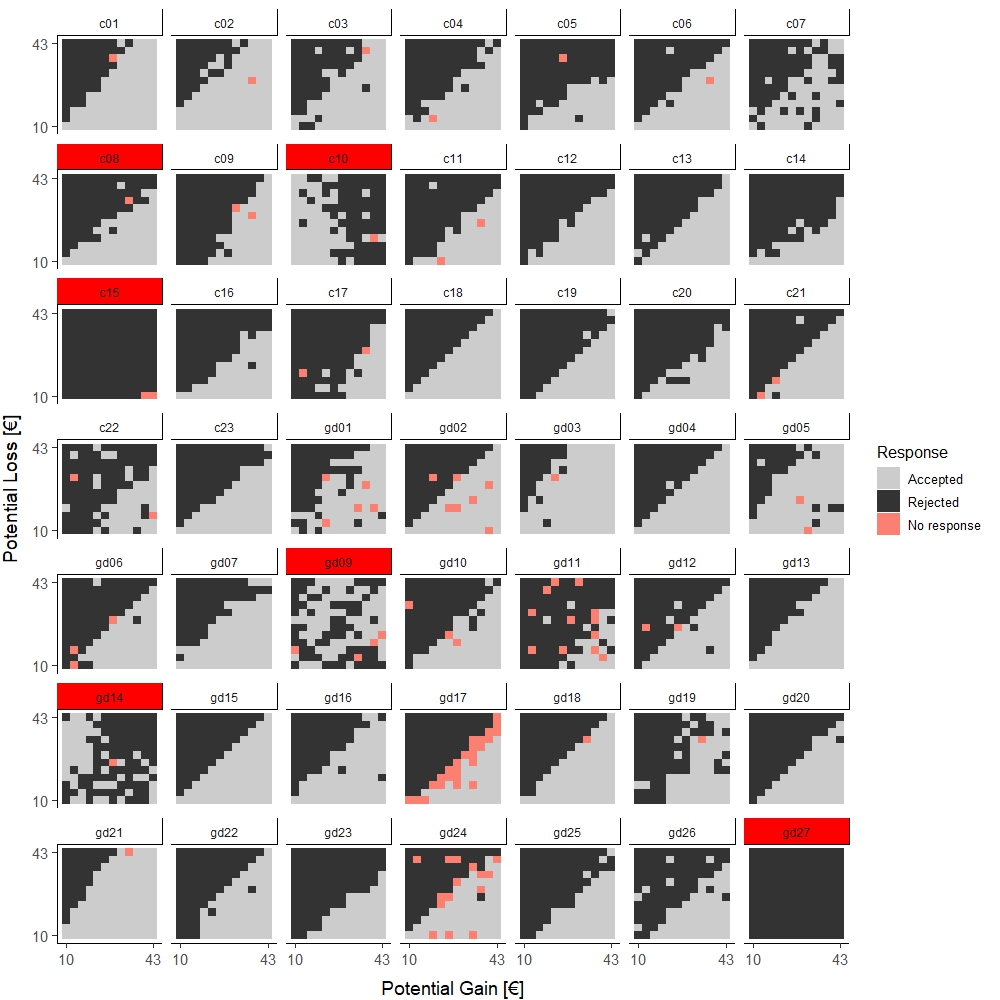

Supplement: Supplementary file 2 — Data S2 Supporting information [file ADB-29-e13396-s002.jpeg]
