# Supplementary material for: Loss, gain and choice difficulty in gambling patients: Neural and behavioural processes
Source: Addict Biol. 2024 May 10;29(5):e13396. doi: 10.1111/adb.13396 (PMC11087675; doi:10.1111/adb.13396)
Supplement: Supplementary file 3 — Figure S1. Individual response patterns for all 49 participants, which initially took part in the study. Because of our exclusion criteria, following participants were excluded from further data analysis: c08 (control with SOGS greater than 4), c15 and gd27 (rejected every gamble ‐unfeasible to calculate a loss aversion parameter), c10, gd09 and gd14 (inexplicable response patterns suggest these participants either did not understand the task or did response unreasonably on purpose) [file ADB-29-e13396-s001.docx]

**Supplementary Material for “Loss, Gain and Choice Difficulty in Gambling Patients: Neural and Behavioural Processes”**

Daniel Freinhofer ^1^, Philipp Schwartenbeck ^1, 2, 3, 4^, Natasha Thon ^5^, Wolfgang Aichhorn ^5^, Melanie Lenger ^1, 6^, Friedrich M. Wurst ^5, 7^, Martin Kronbichler ^1, 2^

^1^ Centre for Cognitive Neuroscience, University of Salzburg, Salzburg, Austria

^2^ Neuroscience Institute, Christian-Doppler Medical Centre, Paracelsus Medical University Salzburg,Salzburg, Austria

^3^ Wellcome Trust Centre for Human Neuroimaging, University College London, London, United Kingdom

^4^ Oxford Centre for Functional MRI of the Brain, Nuffield Department of Clinical Neurosciences, University of Oxford, Oxford, United Kingdom

^5^ Department of Psychiatry, Psychotherapy and Psychosomatics, Christian-Doppler Medical Centre, Paracelsus Medical University, Salzburg, Austria

^6^ Department for Psychiatry and Psychotherapy, Medical University of Graz, Graz, Austria

^7^ Medical Faculty and Psychiatric University Hospital, University Basel, Basel, Switzerland


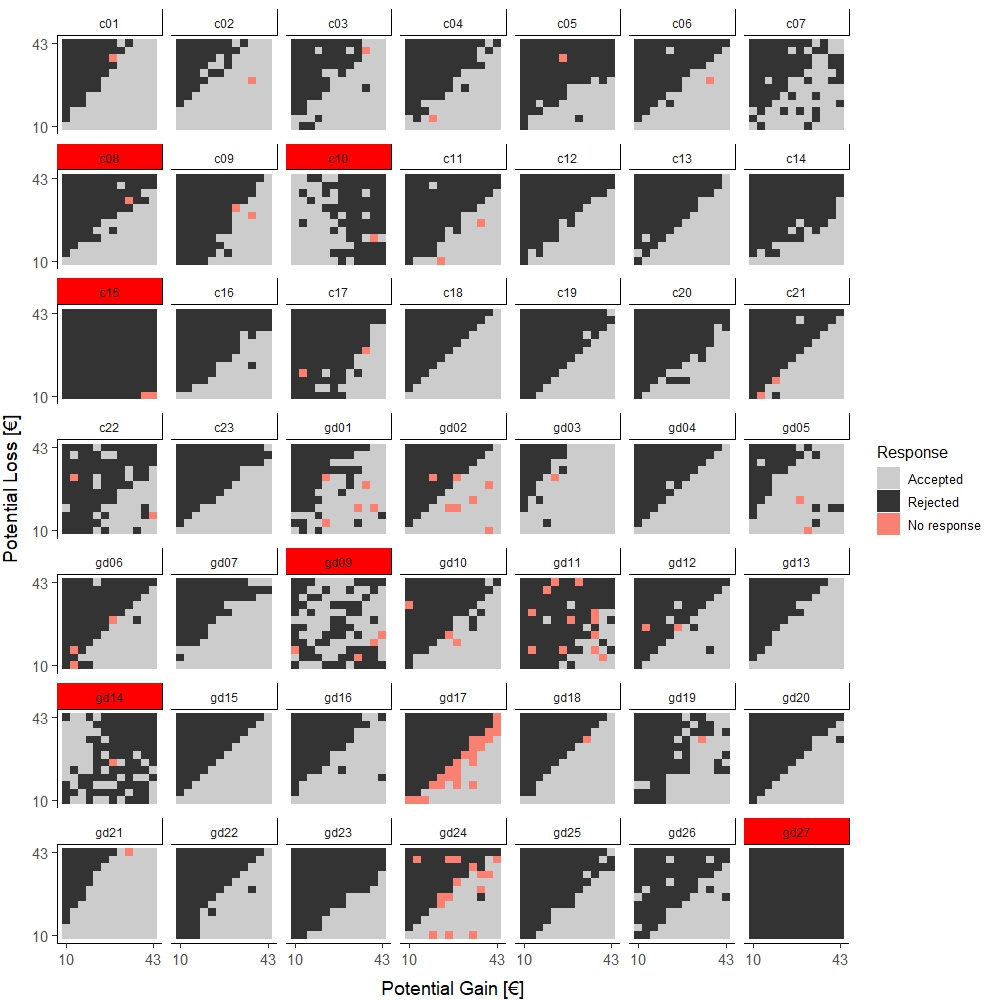


**Figure S1.** Individual response patterns for all 49 participants which initially took part in the

study. Due to our exclusion criteria, following participants were excluded from further data

analysis: c08 (control with SOGS greater than 4), c15 and gd27 (rejected every gamble -

unfeasible to calculate a loss aversion parameter), c10, gd09 and gd14 (inexplicable

response patterns suggest these participants either did not understand the task or did

response unreasonably on purpose)
